# Macronutrient quality index and cardiovascular disease risk in the Seguimiento Universidad de Navarra (SUN) cohort

**DOI:** 10.1007/s00394-022-02901-3

**Published:** 2022-05-22

**Authors:** Paola Vanegas, Itziar Zazpe, Susana Santiago, Cesar I. Fernandez-Lazaro, Víctor de la O, Miguel Ángel Martínez-González

**Affiliations:** 1 School of Pharmacy and Nutrition, Department of Nutrition and Food Sciences and Physiology, University of Navarra, Campus Universitario, 31080 Pamplona, Spain; 2 School of Medicine, Department of Preventive Medicine and Public Health, University of Navarra, Campus Universitario, 31080 Pamplona, Spain; 3 IdiSNA, Instituto de Investigación Sanitaria de Navarra, Pamplona, Navarra Spain; 4 CIBER Fisiopatología de la Obesidad y Nutrición (CIBERobn), Instituto de Salud Carlos III (ISCIII), Madrid, Spain; 5 Department of Nutrition, Harvard T.H Chan School of Public Health, Boston, MA USA

**Keywords:** Macronutrient quality, CVD, Mediterranean diet, Provegetarian diet, Cohort

## Abstract

**Purpose:**

To assess the association between a multi-dimensional Macronutrient Quality Index (MQI) and the risk of cardiovascular disease (CVD) in a Mediterranean cohort.

**Methods:**

Prospective analyses among 18,418 participants (mean age 36 years, 60.8% women) of the Seguimiento Universidad de Navarra (SUN) cohort. Dietary intake information was obtained through a validated semi-quantitative food-frequency questionnaire (FFQ). The MQI (expressing high-quality macronutrient intake) was calculated based on three previously reported quality indices: the Carbohydrate Quality Index (CQI), the Fat Quality Index (FQI), and the Healthy Plate Protein source Quality Index (HPPQI). Adherence to the Mediterranean diet (MedDiet) and Provegetarian Diet was evaluated using the Trichopoulou index and the score proposed by Martínez-González, respectively. CVD was defined as new-onset stroke, myocardial infarction, or CVD death.

**Results:**

After a median follow-up time of 14 years (211,744 person-years), 171 cases of CVD were identified. A significant inverse association was found between the MQI and CVD risk with multivariable-adjusted HR for the highest vs. the lowest quartile of 0.60 (95% IC, 0.38–0.96; *P*_*trend*_ = 0.024).

**Conclusion:**

In this Mediterranean cohort, we found a significant inverse relationship between a multidimensional MQI (expressing high-quality macronutrient intake) and a lower risk of CVD.

## Introduction

Cardiovascular disease (CVD) was the leading cause of death in the world, contributing to around 18 million deaths in 2019 [1]. In Europe, it is estimated that CVD is responsible for one in four deaths [2], accounting for 2.2 million deaths in women and 1.9 million in men [3]. These numbers represent a global health concern and highlight the need for prevention strategies.

Reducing the risk of major modifiable factors such as unhealthy diet, smoking, alcohol consumption, and physical inactivity represents one of the main strategies for the prevention of non-communicable diseases [4, 5]. The American College of Cardiology/American Heart Association (ACC/AHA) emphasizes the importance of healthy dietary patterns rather than their isolated components [6], suggesting a greater magnitude of beneficial effects [7]. Healthy dietary patterns are characterized by high consumption of fruits, vegetables, legumes, whole grains, and fish while limiting the consumption of whole dairy products, red meats, processed meats, and sugars [8]. Diet quality is widely used in nutritional epidemiology to develop national nutrition guidelines, evaluate compliance to predefined healthy dietary patterns, or assess the risk of developing chronic diseases such as CVD [9, 10].

In the last few decades, the dietary approach used for CVD prevention has traditionally focused on diets with low fat intake—particularly on reduction in saturated fatty acids (SFA) and cholesterol—and promotion of high intake of unsaturated fatty acids—such as polyunsaturated fatty acids (PUFA) [11–14]. However, the emerging evidence on fats contradicts such approach [15–18]. Appropriate distribution of macronutrients with respect to total energy intake (45–65%, 10–35%, and 20–35% for carbohydrates, proteins, and fats, respectively), has been associated with lower risk of chronic diseases and adequate micronutrient intake [19]. However, macronutrient quality is likely to be even more important than macronutrient quantity [20, 21].

Most of the existing research on macronutrient quality and CVD has focused on their isolated effects, particularly on fat quality [22, 23], rather than using a more comprehensive approach. To cover this gap, our team created a novel global index, namely the Macronutrient Quality Index (MQI), based on the best scientific evidence available at that moment to evaluate its association with all-cause mortality in the “Seguimiento Universidad de Navarra (SUN)” (University of Navarra Follow-Up) Project [24]. The MQI was constructed upon three equally weighted sub-indexes, one for each macronutrient class (proteins, carbohydrates, and fats), and is an example of an a priori dietary quality index. These indexes are considered useful tools and are widely used in research and clinical practice [25]. To our knowledge, there are no prospective studies that have investigated the association between the dietary quality of macronutrients and the risk of CVD using the MQI. Hence, we aimed to prospectively assess the association of MQI and CVD risk, with a median follow-up time of 14 years, in the SUN cohort.

## Materials and methods

### Study population

**Fig. 1:**
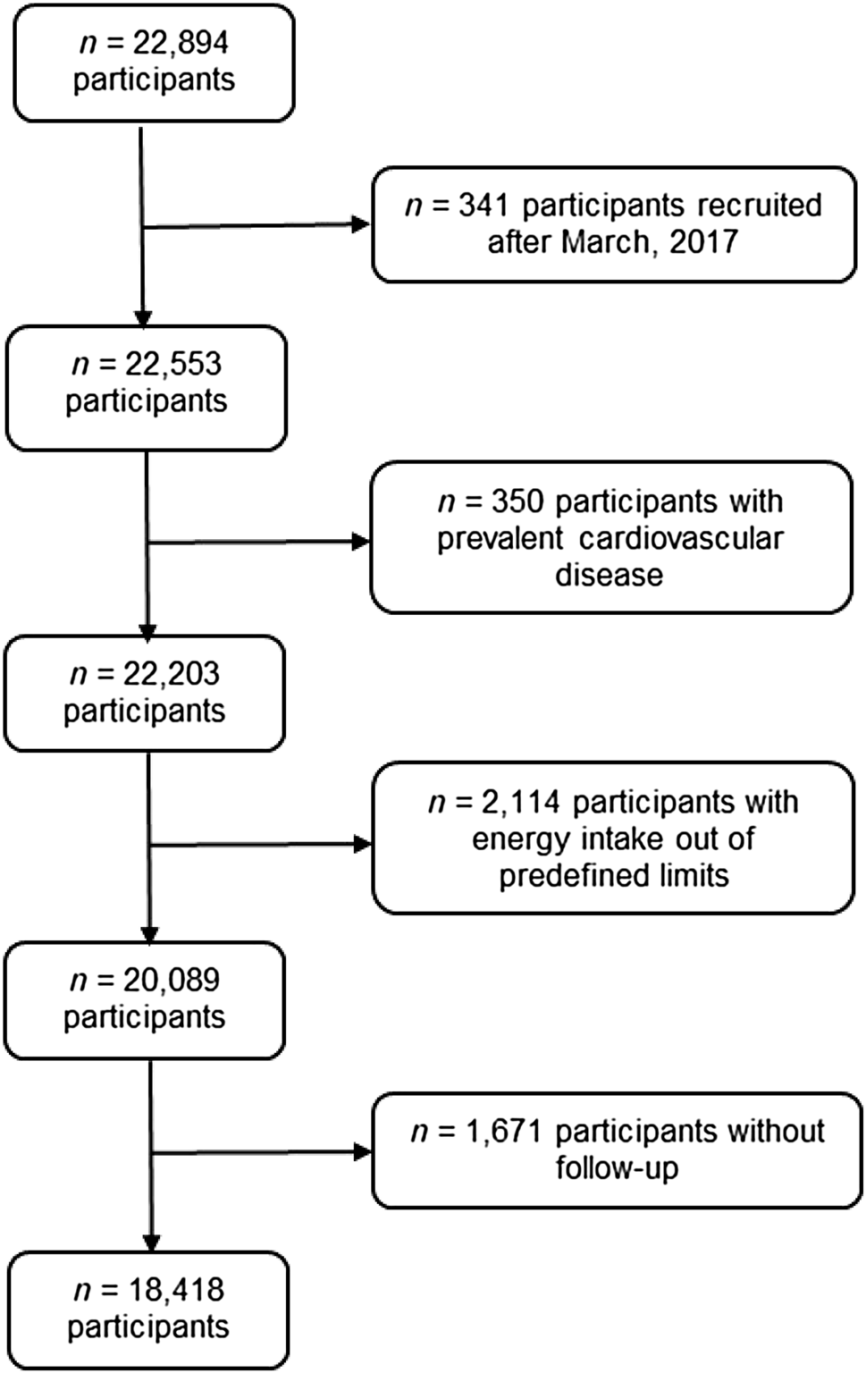
Flowchart of participants included in the study

The SUN Project is a continuous, dynamic, multipurpose and prospective cohort [26]. All participants are university graduates, which reduces the potential confounding related to educational level and socioeconomic status, and increases the validity and reliability of the information [27]. Self-reported mailed and electronic questionnaires are collected at baseline and every 2 years to gather information related to socio-demographics, lifestyle, and medical history, including mortality and its causes. Participants who did not respond to any of the five follow-up mailings were contacted by email or phone. By December 2019, a total of 22,894 participants were enrolled in the SUN cohort. For these analyses, we excluded 341 participants recruited after March 2017 (to ensure a minimum follow-up of 2 years); 350 participants with prevalent CVD; 2,114 individuals with energy intake outside of predefined limits (men: < 800 or > 4,000 kcal/day; women: < 500 or > 3,500 kcal/day) [28]; and 1,671 participants without follow-up (retention rate 91.7%). Therefore, 18,418 participants were the basis for our analyses (Fig. 1).

### Bioethics

Participants received written information about the information collected in the questionnaires, their privacy rights to protect their data, and future feedback of the finding of the project from the research team. Potential candidates were additionally informed about their right to refuse to participate or withdraw from the study at any time without reprisal, according to the ethical standards of the Declaration of Helsinki. Voluntary completion of the baseline questionnaire was considered as informed consent for participation in the study. The Research Ethics Committee of the University of Navarra approved the study. The SUN cohort is registered at clinicaltrials.gov as NCT02669602.

### Dietary assessment

Baseline dietary information and after 10 years of follow-up was evaluated using a self-administered food frequency questionnaire (FFQ). The questionnaire has been previously validated, and the reproducibility for the majority of foods and nutrients is good [29–31]. The FFQ consists of 136 items and includes 9 food groups: (1) dairy products, (2) eggs, meat, and fish, (3) vegetables, (4) fruits, (5) legumes and cereals, (6) oils and fats, (7) pastries, (8) beverages and (9) miscellaneous. For each food, participants reported how often, on average, during the previous year they had consumed, specifying serving size with different options from “never or almost never” to “more than six times a day.”

Spanish food composition tables were used to calculate dietary intake, considering the daily intake of each food and the composition of nutrients [32, 33]. The ad hoc computer system was used to calculate the daily consumption of each food by multiplying the typical serving size by the frequency of consumption.

### MQI assessment

As previously explained, the MQI was constructed based on three sub-indices, the Carbohydrate Quality Index (CQI), the Fat Quality Index (FQI), and the Healthy Plate Protein source Quality Index (HPPQI). The CQI has been used in previous cohort and trial studies to evaluate their association with CVD [34], plasma metabolomic profiles [35], and changes in cardiovascular risk factors [36]. The CQI is based on four equally weighted carbohydrate quality domains: glycemic index (GI), total dietary fiber intake (g/d), ratio of whole grains/ total cereals (whole grains + refined cereals + products prepared with refined flours), and the ratio of solid/total carbohydrates (liquids + solids).

The FQI has been used in nutritional adequacy [37] and CVD investigations [38]. For the calculation of FQI, monounsaturated fatty acids (MUFA), PUFA, SFA, and trans-fat acids (TFA) were taken into account as follows: FQI = (MUFA + PUFA)/(SFA + TFA), receiving equally weighting.

Lastly, the HPPQI has been used in a previous study conducted by our group [24] and it was calculated based on the following ratio: HPPQI = (seafood + poultry + pulses + nuts)/(red and processed meats + cheese), considering the first food group as healthy sources of protein and the second group as unhealthy sources, according to the Harvard’s Healthy Eating Plate [39].

To calculate the MQI, participants were classified into quintiles for each sub-index (CQI, FQI, and HPPQI), assigning values ranging from 1 (lowest quality) to 5 (highest quality). All the sub-index values were summed up, resulting in an MQI score ranging from 3 (poorest macronutrient quality) to 15 (highest macronutrient quality). Lastly, we classified participants into quartiles according to their total MQI score (Table 1).

**Table 1:** Components of the Macronutrient Quality Index (MQI)

Components of the macronutrient quality index	Index range (Points)	Criteria for minimum index	Criteria for maximum index
Carbohydrate quality index (CQI)	1–5	Minimum CQI (first quintile)	Maximum CQI (fifth quintile)
Dietary fiber intake (g/d)	1–5	Minimum dietary fiber intake (first quintile)	Maximum dietary fiber intake (fifth quintile)
Glycemic index	1–5	Maximum glycemic index (fifth quintile)	Minimum glycemic index (first quintile)
Ratio whole grains/(whole grains + refined grains or its products)	1–5	Minimum value of this ratio (first quintile)	Maximum value of this ratio (fifth quintile)
Ratio solid carbohydrates/(solid carbohydrates + liquid carbohydrates)	1–5	Minimum value of this ratio (first quintile)	Maximum value of this ratio (fifth quintile)
Fat quality index (FQI) = (MUFA + PUFA)/(SFA + TFA)	1–5	Minimum FQI (first quintile)	Maximum FQI (fifth quintile)
Healthy plate protein quality index (HPPQI) = = (seafood + poultry + pulses + nuts)/(red and processed meats + cheese)	1–5	Minimum HPPQI (first quintile)	Maximum HPPQI (fifth quintile)
Total MQI (range)	3–15		

### Other dietary scores

Adherence to the Mediterranean diet (MedDiet) was assessed with the well-known score proposed by Trichopoulou et al. [40]. The total score range was from 0 to 9, with higher scores indicating greater adherence.

To assess adherence to the Provegetarian pattern, we used the score proposed by Martínez-González et al. [41]. The total score was calculated by summing up the values of the quintiles of vegetable food (1 point for the lowest quintile and 5 points for the highest quintile) and the values of the quintiles of animal food inversely weighted (1 point for the highest quintile and 5 points for the lowest quintile). The final score ranged from 12 (worst adherence) to 60 points (best adherence) [41].

### Ascertainment of CVD

CVD was the primary endpoint of our study and it was included inquired in by every self-reported follow-up questionnaire collected every 2 years. When the participant reported a CVD event, we requested the medical documentation and a team of cardiologists adjudicated the event, blinded to the dietary exposures. The endpoint was a composite of acute myocardial infarction with or without ST elevation, stroke (both confirmed by a review of medical records with the prior permission of relatives), and cardiovascular death. Cardiovascular events were generally self-reported. Medical records of participants were requested to confirm cases and finally, cardiovascular events were confirmed by a cardiologist who was blind to diet and lifestyle exposure. Additionally, all potential cases were reviewed by a team of expert physicians. Nonfatal stroke was defined as a focal neurological deficit of sudden onset with a duration of more than 24 h and vascular mechanism. Diagnosis of myocardial infarction was defined using universal criteria [42]. Deceases from cardiovascular causes were confirmed by death certificates, medical records, or records linked to the National Institute of Statistics. For participants lost during follow-up, we consulted the National Death Index of Spain at least once a year, to identify any member of the cohort who may have died.

### Other covariates

Additional covariates include anthropometric measurements, habits related to health and lifestyle. The validity of self-reported anthropometric information (weight and height) has been previously evaluated in a subsample of the SUN cohort [43].

### Statistical analysis

We describe the baseline characteristics of participants adjusted for age and sex using the inverse probability weighting method according to quartiles of the MQI. Proportions for categorical variables and means and standard deviation (SD) for quantitative variables were calculated.

Cox proportional hazard regression models were used to estimate the association between the quartiles of MQI and CVD incidence. Hazard ratios (HRs) were calculated with their 95% confidence intervals (CIs) for each quartile, considering Q1 as the reference category. The interpretation of HR > 1 was considered a higher CVD risk, whereas HR < 1 was considered a lower probability of CVD.

Based on the existing literature and also on previous findings of the SUN cohort on [44, 45], we adjusted our models as follows: age was used as underlying time-variable in all models; model 1 was adjusted for sex, age (deciles), and stratified by year entering the cohort; model 2 was additionally adjusted for total energy intake (kcal/d, continuous), marital status (single, married, widowed, separated and others), educational level (years of higher education, continuous), smoking (never, current, and former smoker), accumulated smoking habit (pack-years, continuous), alcohol intake (never, < 5 women or < 10 men g/d, 5–25 women or 10–50 men g/d, and > 25 women or > 50 men g/d), physical activity (metabolic equivalent-h/week, continuous), snacking between meals (yes/no), body mass index (BMI [kg/m^2^, linear and quadratic terms, continuous]), time spent sitting (hours/week, continuous), weight gain in the previous 5 years before entering the cohort (< 3 kg and ≥ 3 kg) and following a special diet at baseline (yes/no); model 3, was additionally adjusted for family history of CVD (yes/no), and any diagnosis of diabetes (yes/no), hypertension (yes/no), hypercholesterolemia (yes/no), dyslipidemia (yes/no), depression (yes/no), cancer (yes/no); and lastly, model 4 was adjusted for total carbohydrate intake (g/d, continuous), total fat intake (g/d, continuous), and total protein intake (g/d, continuous).

Linear trend tests were performed through successive quartiles, assigning the median value of each quartile, and treating the resulting variables as continuous.

To minimize any effect of dietary variation, we used repeated measurements with updated data and cumulative diet average information of the MQI and its components, with a complete repetition of the FFQ after 10 years of follow-up. For the analysis of repeated measures, the mean between the baseline FFQ and the 10 year FFQ (i.e., cumulative average exposure) was calculated to assess a more realistic diet based on the MQI.

We additionally evaluated the combined effects of adherence to the MedDiet and the Provegetarian dietary pattern with the MQI. For both indexes, participants were categorized into two groups (below and above the median), interpreted as “low adherence” and “high adherence”, respectively, while the MQI was categorized into three groups (Q1, Q2-Q3, and Q4). We considered as reference category the Q4 of the MQI and the highest adherence to MedDiet or Provegetarian dietary pattern.

The following sensitivity analyses and subgroup analyses were additionally performed to assess the robustness of our findings: (a) selection by sex, only men or women participants, (b) only participants < 45 years or ≥ 45 years, (c) censoring participants at > 50 years, (d) only health professionals or only non-health professionals participants, (e) exclusion of participants with hypercholesterolemia and prevalent hypertension, (f) using different predefined energy intake limits (5th percentile and 95th percentile), (g) exclusion of participants with prevalent cancer, (h) exclusion of participants who followed a special diet at baseline, (i) exclusion of participants with ≥ 30 items missing in the FFQ, and (j) exclusion of participants with early CVD (≤ 2 years).

Finally, the Nelson-Aalen curves were used to represent the cumulative risk of CVD during the follow-up of the study according to tertiles of MQI (T1: < 8, T2:8–10 y T3 ≥ 11).

Statistical analyses were conducted using STATA version 16 (STATA Corporation) with the SUN database updated in December 2019. All *p* value were two-tailed, and statistical significance was deemed in the conventional cut-off *p* < 0.05.

## Results

### Baseline characteristics of the participants

A total of 18,418 participants were followed for a mean time of 14 years (211,744 person-years). During this time, 171 cases of prevalent CVD were identified, including 82 cases of nonfatal acute myocardial infarction, 61 cases of nonfatal strokes, and 28 CV exitus.

Table 2 presents the characteristics of the participants, according to the quartiles of the MQI adjusted for age and sex.

**Table 2:** Age and sex-adjusted baseline characteristics using the inverse probability weighting according to quartiles of the MQI among participants in the SUN cohort (*n* = 18,418)

	Q1	Q2	Q3	Q4
*n*	6,408	4,363	3,768	3,879
MQI range	3–7	8–9	10–11	12–15
MQI (median)	6	9	10	13
Marital status (%)				
Single	43.2	43.7	43.7	46.2
Married	51.9	50.5	50.7	47.4
Widowed	0.9	1.1	1.0	0.8
Separated	2.2	2.3	2.4	3.1
Others	1.9	2.4	2.3	2.4
Years of university education	5.1 (1.5)	5.1 (1.5)	5.0 (1.5)	5.0 (1.5)
Health professionals (%)	61.0	63.9	66.4	68.9
Smoking (%)				
Never smoker	48.1	47.2	50.7	50.8
Current smoker	23.6	24.7	20.1	17.8
Former smoker	28.3	28.2	29.2	31.4
Cumulative smoking habit (pack-years)	5.9 (9.9)	5.9 (9.6)	5.4 (9.3)	5.3 (8.8)
Alcohol intake (g/d)				
Never	18.6	17.3	17.4	18.0
< 5 women/ < 10 men	48.4	48.6	48.3	49.8
5–25 women/10–50 men	31.1	32.0	32.2	30.9
> 25 women/ > 50 men	1.8	2.2	2.1	1.3
Physical activity (METs-h/week)	19.1 (20.7)	20.7 (21.8)	22.9 (24.5)	26.4 (25.9)
BMI (kg/m^2^)	23.4 (3.5)	23.6 (3.5)	23.7 (3.6)	23.4 (3.4)
Time spent sitting (h/d)	5.4 (2.1)	5.3 (2.1)	5.2 (2.0)	5.2 (2.0)
Family history of CVD (%)	13.6	14.3	13.7	13.6
Medically-diagnosed condition at baseline (%)				
Diabetes	1.5	1.8	2.2	2.1
Hypertension	9.7	10.1	10.9	11.5
Hyperchoresterolemia	14.6	16.2	19.0	19.1
Dyslipemia	5.9	6.5	7.4	7.2
Cancer	2.6	2.3	2.4	2.7
Depression	10.8	11.3	11.8	12.5
Snacking between meals (%)	35.3	34.4	31.7	29.7
Special diet (%)	5.1	7.0	8.9	14.2
Supplementation (%)	17.2	17.5	19.9	22.5
MedDiet score^a^	3.0 (1.4)	4.1 (1.4)	4.9 (1.4)	5.9 (1.4)

[Means (SD) or percentages] *%* percentage, *Q* quartile, *BMI* body mass index, *METs-h*, metabolic equivalent-h/week; Values are means ± SD or percentages of the number of participants under otherwise indicated

^a^MedDiet: according to the score proposed by Trichopoulou et al (31)

The mean age of the participants was 36 (SD 12.1) years, and the mean baseline BMI was 23.1 (SD 3.5) kg/m^2^. Around 61% of the participants were women. Participants in the highest quartile of the MQI were more likely to be single, healthcare professionals, physically active, and less likely to snack between meals. Additionally, participants with better MQI were more likely to follow a special diet, consume some type of supplementation, and have greater adherence to the MedDiet.

Regarding the dietary characteristics of participants, those with higher values of MQI consumed more vegetables, fruits, legumes, whole grains, fish, white meats, skimmed dairy products, nuts, and olive oil, but smaller quantities of whole dairy, eggs, soft drinks, and fast food. Regarding the baseline intake, participants in the highest quartile had a higher proportion of energy intake from carbohydrates, PUFA, and showed greater fiber intake, while their proportion of total fat, SFA, TFA, and cholesterol intake was lower (Table 3).

**Table 3:** Age and sex-adjusted dietary baseline characteristics using inverse probability weighting according to quartiles of the MQI among participants in the SUN cohort (*n* = 18,418)

	Q1	Q2	Q3	Q4
*n*	6,408	4,363	3,768	3,879
MQI range	3–7	8–9	10–11	12–15
MQI (median)	6	9	10	13
MQI range	3–7	8–9	10–11	12–15
Food (g/d)				
Vegetables	399 (231)	503 (299)	583 (342)	718 (408)
Fruits	255 (205)	322 (258)	379 (309)	482 (367)
Legumes	18 (11)	22 (16)	25 (19)	28 (25)
Cereals	96 (71)	101 (72)	103 (72)	109 (74)
Whole grains	4.2 (16)	9.4 (25)	15 (33)	30 (44)
Refined grains	90 (53)	90 (52)	88 (52)	78 (49)
Fish	75 (43)	95 (52)	108 (59)	128 (76)
Meats	191 (76)	182 (78)	168 (75)	144 (74)
White	38 (30)	47 (33)	51 (38)	54 (40)
Red	93 (46)	81 (46)	69 (41)	51 (35)
Whole dairy	260 (227)	193 (181)	155 (160)	111 (125)
Skimmed dairy	209 (249)	221 (246)	241 (239)	261 (256)
Eggs	24 (16)	23 (15)	22 (14)	21 (15)
Nuts	4.5 (6.1)	5.8 (8.1)	7.8 (11)	13 (18)
Olive oil	14 (11)	18 (15)	20 (15)	23 (16)
Beverages	71 (137)	63 (114)	62 (125)	53 (120)
Fast-food	23 (21)	22 (20)	20 (20)	17 (18)
Energy and nutrients				
Energy (kcal/d)	2,305 (609)	2,355 (617)	2,374 (633)	2,366 (618)
Carbohydrates (% TEI)	41(7.3)	43 (7.1)	43 (7.1)	45(7)
Fiber (g/d)	21 (8.1)	26 (10)	30 (11)	37 (13)
Proteins (% TEI)	18 (3.3)	18 (3.1)	18 (3.5)	18 (3.5)
Fats (% TEI)	37 (6.1)	36 (6.3)	35 (6.6)	34 (7)
MUFAs	15 (3.2)	15 (3.7)	15 (4.0)	15 (4.1)
PUFAs	4.8 (1.4)	5.2 (1.5)	5.3 (1.6)	5.4 (1.6)
SFAs	14 (3.0)	12 (2.5)	11 (2.4)	9.7 (2.4)
TFAs	0.5 (0.2)	0.4 (0.1)	0.3 (0.1)	0.2 (0.1)
Cholesterol (mg/d)	435 (148)	425 (151)	406 (142)	372 (143)

% *TEI* percentage of total energy intake, *PUFA* polyunsaturated fatty acids, *MUFA* monounsaturated fatty acids, *SFA* saturated fatty acids, *TFA* trans fatty acids

### Association between MQI and CVD

Table 4 shows the results of the multivariate Cox regression analysis for the association between MQI and CVD risk. An inverse association between the MQI and the risk of CVD was observed in all models. Point estimates monotonically decreased across successive quartiles of MQI. In the fully adjusted model, the relative risk of CVD was 40% lower for participants in the highest quartile when compared with the lowest quartile, with HR of 0.60 (95% CI, 0.38 – 0.96, *P*_*trend*_ = 0.024).

**Table 4:** Hazard ratios (HR) and 95% confidence intervals (CI) for the association between MQI and the risk of cardiovascular disease in 18,418 participants in the SUN cohort

	Q1	Q2	Q3	Q4	*p* for trend
*n* (frequency)	6,408	4,363	3,768	3,879	
MQI range (min–max)	3–7	8–9	10–11	12–15	
CVD	62	42	33	34	
Person-years	76,374	50,675	42,586	42,107	
Mortality rate/1000 person years	0.81	0.83	0.77	0.81	
Model 1	1.00 (Ref.)	0.86 (0.58–1.28)	0.73 (0.47–1.11)	0.62 (0.40–0.95)	0.022
Model 2	1.00 (Ref.)	0.87 (0.58–1.31)	0.73 (0.47–1.13)	0.65 (0.42–1.02)	0.050
Model 3	1.00 (Ref.)	0.91(0.61–1.36)	0.73 (0.47–1.14)	0.63 (0.40–1.00)	0.039
Model 4	1.00 (Ref.)	0.86 (0.58–1.30)	0.70 (0.45–1.09)	0.60 (0.38–0.96)	0.024

Model 1 was adjusted for sex, age (deciles), and stratified by year entering the cohort; model 2 was additionally adjusted for total energy intake (kcal/d, continuous), marital status (single, married, widowed, separated and others), educational level (years of higher education, continuous), smoking (never, current, and former smoker), accumulated smoking habit (pack-years, continuous), alcohol intake (never, < 5 women or < 10 men g/d, 5–25 women or 10–50 men g/d, and > 25 women or > 50 men g/d), physical activity (metabolic equivalent-h/week, continuous), snacking between meals (yes/no), body mass index (BMI [kg/m2, linear and quadratic terms, continuous]), time spent sitting (hours/week, continuous), weight gain in the previous 5 years before entering the cohort (< 3 kg and ≥ 3 kg) and following a special diet at baseline (yes/no); Model 3, was additionally adjusted for family history of CVD (yes/no), and any diagnosis of diabetes (yes/no), hypertension (yes/no), hypercholesterolemia (yes/no), dyslipidemia (yes/no), depression (yes/no), cancer (yes/no); lastly, model 4 was adjusted for total carbohydrate intake (g/d, continuous), total fat intake (g/d, continuous), and total protein intake (g/d, continuous)

*Ref* referent value

To have an updated dietary approach, we conducted time-dependent Cox regression models with repeated measurements of dietary exposures using cumulative average information after a 10 year follow-up. Despite all models revealed an inverse association between the MQI and the risk of CVD, none of the models reached statistical significance, with HR of 0.68 (95% CI 0.42–1.09, *P*_*trend*_ = 0.101) for updated dietary information and HR of 0.65 (95% CI 0.41–1.03, *P*_*trend*_ = 0.073) when comparing participants in the highest *vs* the lowest quartile in fully adjusted models (Table 5).

**Table 5:** Repeated nutritional measures. Association between quartiles of MQI and CVD risk in 18,418 participants of the SUN Cohort

^1^Updated Diet	Q1	Q2	Q3	Q4	*p* for trend
MQI Range (min–max)	3–6	7–9	10–11	12–15	
CVD	47	55	31	38	
Person-years	54,165	74,717	41,265	41,596	
Mortality rate/1000 person-year	0.87	0.74	0.75	0.91	
Model 1	1.00 (Ref.)	0.75 (0.50–1.12)	0.67 (0.42–1.07)	0.68 (0.44–1.07)	0.086
Model 2	1.00 (Ref.)	0.76 (0.50–1.14)	0.69 (0.43–1.11)	0.73 (0.46–1.16)	0.170
Model 3	1.00 (Ref.)	0.79 (0.52–1.19)	0.70 (0.43–1.13)	0.71 (0.45–1.14)	0.146
Model 4	1.00 (Ref.)	0.75 (0.50–1.13)	0.66 (0.41 – 1.07)	0.68 (0.42–1.09)	0.101

^2^ Cumulative Diet Average	Q1	Q2	Q3	Q4	p for trend
MQI Range (min–max)	3–7	7.5–9	9.5–11	11.5–15	
CVD	62	39	35	35	
Person-years	76,416	51,615	42,543	41,167	
Mortality rate/1000 person-year	0.81	0.76	0.82	0.85	
Model 1	1.00 (Ref.)	0.78 (0.52–1.17)	0.78 (0.51–1.19)	0.66 (0.43 – 1.01)	0.063
Model 2	1.00 (Ref.)	0.79 (0.52–1.19)	0.80 (0.52–1.23)	0.70 (0.45 – 1.10)	0.133
Model 3	1.00 (Ref.)	0.82 (0.54–1.23)	0.81 (0.52–1.24)	0.68 (0.44 – 1.07)	0.104
Model 4	1.00 (Ref.)	0.78 (0.52–1.18)	0.77 (0.50–1.19)	0.65 (0.41 – 1.03)	0.073

Using two approaches: updated diet^1^ and cumulative diet average^2^ at baseline and after 10 years of follow-up. Hazard ratios (HR) and 95% confidence intervals (CI)

^1^Repeated Measures: update information of MQI after 10 years of follow-up. ^2^Repeated Measures: cumulative average information of MQI at baseline and after 10 years of follow-up. Model 1 was adjusted for sex, age (deciles), and stratified by year entering the cohort; model 2 was additionally adjusted for total energy intake (kcal/d, continuous), marital status (single, married, widowed, separated and others), educational level (years of higher education, continuous), smoking (never, current, and former smoker), accumulated smoking habit (pack-years, continuous), alcohol intake (never, < 5 women or < 10 men g/d, 5–25 women or 10–50 men g/d, and > 25 women or > 50 men g/d), physical activity (metabolic equivalent-h/week, continuous), snacking between meals (yes/no), body mass index (BMI [kg/m2, linear and quadratic terms, continuous]), time spent sitting (hours/week, continuous), weight gain in the previous 5 years before entering the cohort (< 3 kg and ≥ 3 kg) and following a special diet at baseline (yes/no); Model 3, was additionally adjusted for family history of CVD (yes/no), and any diagnosis of diabetes (yes/no), hypertension (yes/no), hypercholesterolemia (yes/no), dyslipidemia (yes/no), depression (yes/no), cancer (yes/no); lastly, model 4 was adjusted for total carbohydrate intake (g/d, continuous), total fat intake (g/d, continuous), and total protein intake (g/d, continuous)

*Ref* referent value

### The combined analyses of MQI and other dietary scores and the incidence of CVD

**Fig. 2:**
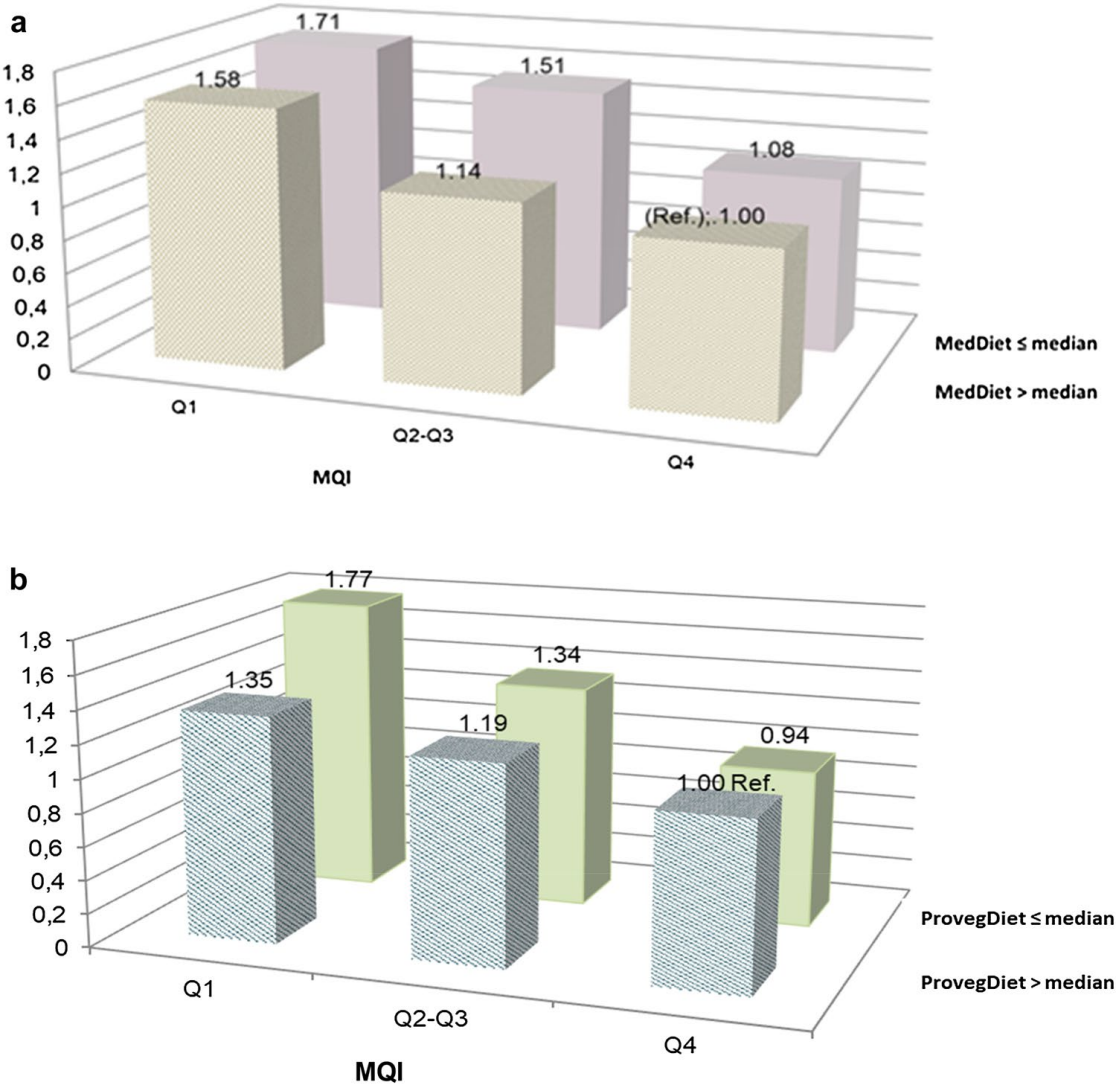
**a** Hazard ratios (HR) and confidence intervals (CI) for CVD risk according to the combined effect of the MQI categories and the grade of adherence to the MedDiet in 18,418 participants of the SUN cohort*. **b** Hazard ratios (HR) and confidence intervals (CI) for CVD risk according to the combined effect of the MQI categories and the grade of adherence to a provegetarian pattern in 18,418 participants of the SUN cohort*. *Adjusted for sex, age (deciles), and stratified by year entering the cohort, total energy intake, marital status (five categories), educational level, smoking (three categories), accumulated smoking habit, alcohol intake, physical activity, snacking between meals (yes/no), BMI, sitting time, weight gain in the previous 5 years before entering the cohort (< 3 kg and  ≥ 3 kg), following a special diet at baseline, family history of CVD (yes/no), any diagnosis of diabetes (yes/no), hypertension (yes/no), hypercholesterolemia (yes/no), dyslipidemia (yes/no), depression (yes/no), cancer (yes/no), total carbohydrate intake, total fat intake, and total protein intake

Figure 2a and b represent HRs for the incidence of CVD according to the combined analysis of MQI and adherence to MedDiet and Provegetarian Diet, respectively. All participants were categorized into six groups according to the quartile of the MQI (3 groups) and adherence to MedDiet and Provegetarian Diet (two groups, below and above the median).

Overall, for participants with lower adherence to the MedDiet (Fig. 2a) and Provegetarian Diet (Fig. 2b), the risk of CVD increased across higher categories of the MQI. Thus, the HR (95% CI) for participants with lower adherence to the MedDiet (≤ median) and lower MQI (Q1) was 1.71 (1.06—2.77) as compared to participants with higher adherence to the MedDiet (> median) and higher MQI (Q4). Similar results were observed for the analyses of the joint exposure to MQI and adherence to the Provegetarian Diet (Fig. 2b). Participants with lower adherence to both the MedDiet (≤ median) and lower MQI (Q1) had a HR (95% CI) of 1.77 (1.07—2.94).

### Sensitivity analyses

**Fig. 3:**
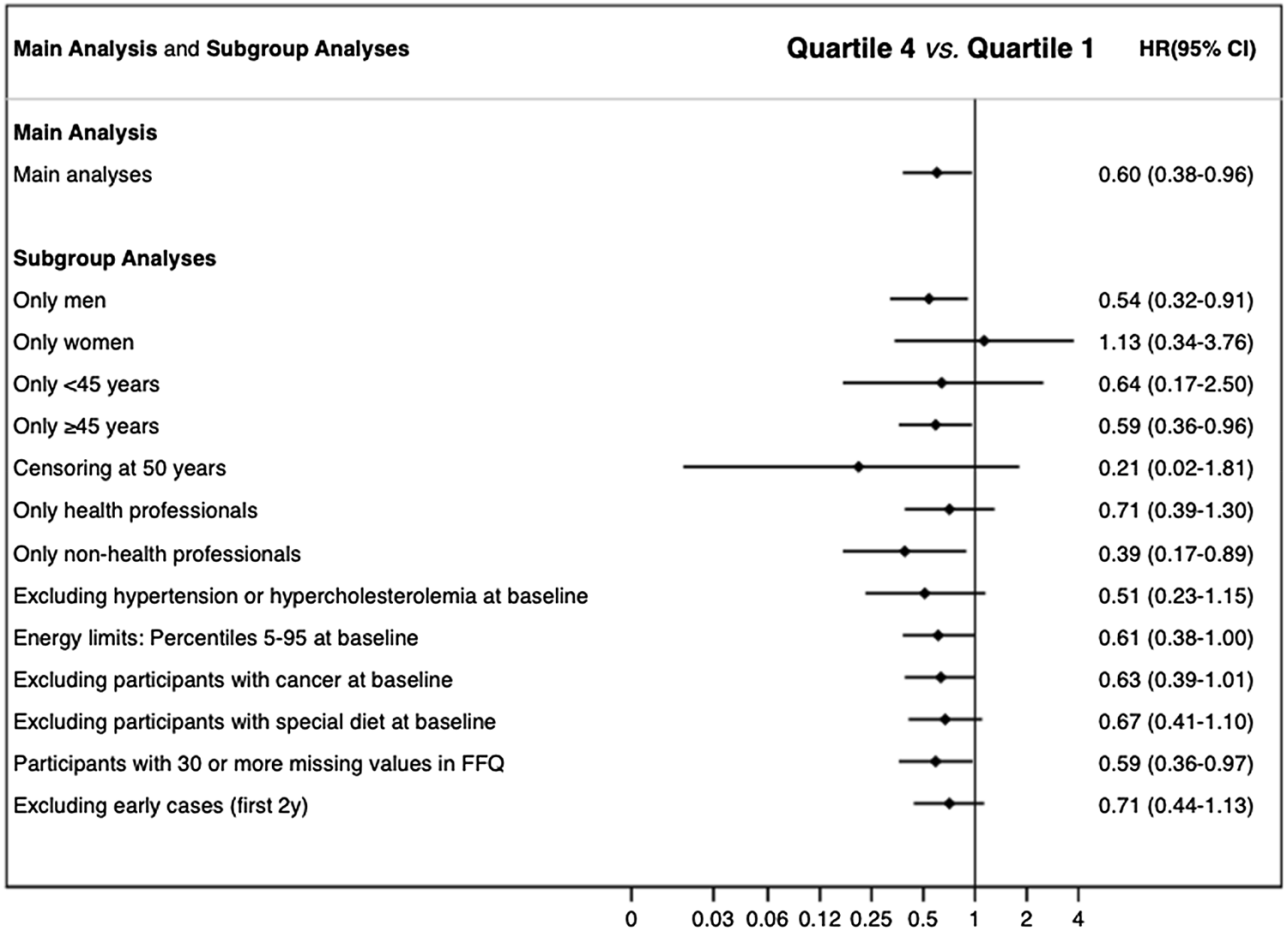
Hazard ratios (HR) and 95% confidence intervals (CI) for the association between MQI and the risk of CVD in the SUN cohort. Quartile 4 vs Quartile 1. Adjusted for sex, age (deciles), and stratified by year entering the cohort, total energy intake, marital status (five categories), educational level, smoking (three categories), accumulated smoking habit, alcohol intake, physical activity, snacking between meals (yes/no), BMI, sitting time, weight gain in the previous 5 years before entering the cohort (< 3 kg and ≥ 3 kg), following a special diet at baseline, family history of CVD (yes/no), any diagnosis of diabetes (yes/no), hypertension (yes/no), hypercholesterolemia (yes/no), dyslipidemia (yes/no), depression (yes/no), cancer (yes/no), total carbohydrate intake, total fat intake, and total protein intake

Multiple sensitivity analyses were performed to corroborate our findings (Fig. 3). Overall, the results did not substantially change in any scenario or sub-group, observing an inverse association between MQI and the incidence of CVD. Point estimates were consistent with the HRs observed in the main analysis, except when men were excluded (HR = 1.13, 95% CI 0.34–3.76).

**Fig. 4:**
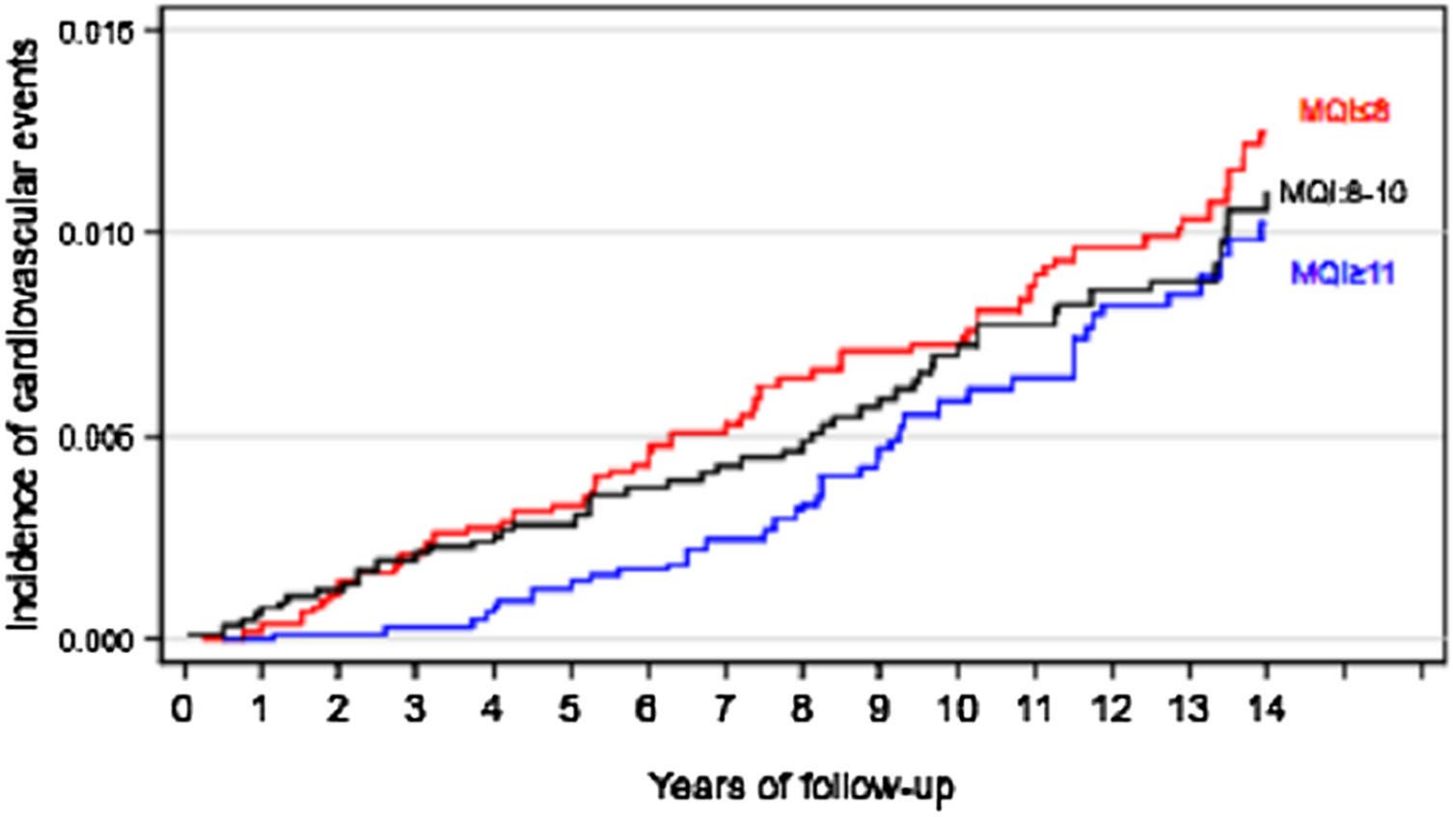
Nelson-Aalen estimate of cumulative risk of CVD in relation to years of follow-up and tertiles of the MQI in the SUN cohort (adjusted for confounding using inverse probability weighting)

We used the Nelson-Aalen survival curve to graphically represent the association between CVD incidence and MQI during the follow-up of the study after controlling for confounding using inverse probability weighting (Fig. 4).

## Discussion

Diet is a major determinant of health and life expectancy. Quality diets should recommend appropriate amounts and proportions of nutrient intake to help educate population about healthy choices. In the past years, a strong interest has emerged to design tools to assess the quality of the diet. The present study is the first to prospectively investigate the association between a novel and multidimensional MQI and the incidence of CVD. Unlike other previous a priori indices, this MQI does not assess adherence to an eating pattern or dietary guideline, but it specifically appraises the global macronutrient quality and quantity. Thus, the Pearson’s correlation coefficient between the MQI and MedDiet was 0.65.

As expected, participants with better MQI were healthier—had higher intakes of vegetables, fruits, legumes, whole grains, fish and seafood, white meats, skimmed dairy products, nuts, olive oil, PUFA, and fiber. These food groups contain high nutritional density and contain bioactive compounds such as flavonoids, polyphenols, and oleocanthal that confer cardioprotective effects [11, 13]. Moreover, the consumption of these food groups provides additional functional properties such as low energy density and low glycemic, which may reduce the risk of CVD [46–48]. Several studies have found that preference for whole grains over refined grains was associated with lower CVD risk. Whole grains preserve all the components (the bran, germ, and endosperm), which help decrease total cholesterol, LDL, body fat, and maintain adequate levels of postprandial glucose [49–52]. A previous study in this cohort demonstrated an inverse association between better carbohydrate quality or a higher proportion of energy from better quality carbohydrates and the risk of CVD and a higher proportion of carbohydrates from whole grains was strongly inversely associated with CVD. Moreover, the study suggested that replacing the amount of refined cereals with the same amount of whole grains and replacing bakery products or cookies with whole bread, the risk of CVD was significantly reduced [34].

Dietary fat has been considered one of the most important modifiable factors associated with the risk of CVD [53]. The traditional recommendations to prevent CVD have been focused on SFA [22]. Currently, the Dietary Guidelines for Americans (DGA) suggest considering fats as part of healthy dietary patterns [12]. In our study, we found participants with higher MQI showed a higher percentage of energy coming from PUFA and a lower percentage of energy from SFA. This is consistent with previous findings of multiple studies in which lower intake of SFA and higher intake of MUFA or PUFA from fish, nuts, and vegetable oils such as olive oil, were associated with a lower incidence rate of CVD [22, 53–55]. The lower incidence rate of CVD may be explained by the consequent decrease in LDL, which is well known to be the main causal factor in the development of atherosclerosis [56]. Previous research in the SUN cohort assessed the dietary quality of fats using the FQI. This study did not find any association between a better fat quality diet and the risk of incident CVD. The authors concluded that a “heart-healthy diet” should focus on a general dietary pattern approach rather than limiting total fat intake or certain subtypes of fat [38].

In our study, we found that participants with higher consumption of red meat, whole dairy products, and eggs, had a lower MQI. However, epidemiological evidence of the long-term effects of higher protein intake on CVD is not clear [57]. Previous findings have demonstrated that intake of vegetable protein from legumes, fruits, vegetables, and nuts was associated with a lower risk of CVD. Conversely, vegetable protein intake from cereals and potatoes was not associated with all-cause mortality and CVD [58, 59]. Animal protein, mainly red meat, has a high content of SFA that increases plasma concentrations of LDL cholesterol [58, 59], which is linked to the etiopathogenesis of CVD [17]. Therefore, the replacement of red meat by fish or poultry and the increase in the consumption of plant protein may produce positive cardioprotective effects [57]. Additionally, changing the proportion of animal protein by vegetable protein may lead to greater CVD risk reduction due to the lower energy density of these foods, and consequently, a reduction in body weight, a major modifiable factor involved in the incidence of CVD [57].

In this sense, Michas et al. [60] suggested that the total matrix of food is more important than just the content of certain nutrients to predicting the effect on CVD risk. Thus, recommendations to improve the dietary quality should be oriented towards dietary patterns that have an adequate contribution of macronutrients, such as the MedDiet, and should not focus on isolated nutrients [61].

The benefits of MedDiet in the prevention of CVD have been reported in multiple studies [62]. Strong and robust evidence exists about the beneficial effects of the MedDiet on the prevention of chronic diseases, particularly CVD due to its high content of legumes, fruits, vegetables, nuts, fish, and olive oil as the main fat, and its low content of meat products [62, 63].

The cardioprotective effects of the provegetarian pattern may be explained due to the high content of foods from plants, fruits, fiber, PUFA, lower intake of animal foods such as red and processed meats, and SFA. These foods lead to positive outcomes such as reduction in lower blood pressure, LDL, inflammatory processes, and improvements in glycemic control [64]. This is supported by a recent meta-analysis with seven studies and 15,077 cases of CVD, in which the authors concluded that greater adherence to the plant-based diet conferred CVD protection [65]. However, it is important to highlight that unhealthy plant-based diets may be associated with higher CVD risk. Lower intakes of unsaturated fats, micronutrients, antioxidants, dietary fiber, and higher intakes of added sugar and glycemic load may explain this detrimental association [66, 67].

Some limitations of the present study should be noted. First, our participants had a high level of education, and results may be considered non-representative of the general population. However, the generalizability of results should be based on biological mechanisms rather than statistical representativeness. Second, the FFQ was self-reported, which may lead to measurement errors. Nevertheless, the FFQ is a gold standard tool in nutritional epidemiology for assessing eating habits and has been repeatedly validated [23–25]. Third, residual confounding cannot be excluded, despite our models were adjusted for traditional CVD risk factors. Fourth, it has not been possible to independently evaluate the association of MQI with fatal cardiovascular disease, due to the small number of fatal events (only 28) in our cohort, that precluded component-specific analyses for the composite CVD outcome. Finally, the MQI has not been formally validated to date. Although some food groups with cardio-protective properties were correlated with the MQI, the novelty of our findings is the more comprehensive nature of our approach that goes beyond assessing the amounts or proportions of each macronutrient in a particular diet and considers instead the overall quality of the three major macronutrients. In addition, this index is based on the best scientific evidence and it was previously used in other studies [24].

The strengths of the study rely on its large sample size, the large follow-up period, the high retention rate (> 91%), the ability to control for a wide number of potential confounders, the blind confirmation of cardiovascular events by medical records which minimizes the potential misclassification bias, the use of repeated measurements of diet after 10 years of follow-up, the numerous sensitivity analyses, and the confirmation of deaths by the Spanish National Death Index.

## Conclusions

In conclusion, in this Mediterranean cohort, we found a significant inverse relationship between a multidimensional MQI and a lower risk of CVD. Additionally, we found that better MQI with greater adherence to MedDiet or the Provegetarian diet was also associated with a lower risk of CVD.

More research is needed to establish appropriate dietary guidelines about the quality of macronutrients to reduce CVD risk based on healthy dietary patterns such as the Mediterranean or the Provegetarian.
